# Falls among community-dwelling older adults in Ethiopia; A preliminary cross-sectional study

**DOI:** 10.1371/journal.pone.0221875

**Published:** 2019-09-10

**Authors:** Balamurugan Janakiraman, Melaku Hailu Temesgen, Gashaw Jember, Asmare Yitayeh Gelaw, Berihu Fisseha Gebremeskel, Hariharasudhan Ravichandran, Emnet Worku, Yohannes Abich, Fekadu Yilak, Misganaw Belay

**Affiliations:** 1 Department of Physiotherapy, School of Medicine College of Medicine and Health Sciences, University of Gondar and Gondar University Specialized Comprehensive Hospital, Gondar, Ethiopia; 2 Department of Physiotherapy, School of Medicine, College of Health Sciences and Ayder Comprehensive Specialized Hospital, Mekelle University, Mekelle, Ethiopia; 3 Department of Epidemiology and Preventive Medicine, Monash University, Melbourne, Victoria, Australia; Catholic University of Korea College of Medicine, REPUBLIC OF KOREA

## Abstract

**Background:**

Falls among older adults is a common precipitating factor for unintentional injuries and represent a major health problem associated with increased morbidity, mortality, and health care cost in low-and-middle-income countries. The burden of fall in this population is well established in high-income countries and scant attention is given to this precipitating factor in low-and-middle-income countries, including Ethiopia. Therefore, this study aimed to estimate the prevalence and factors associated with fall among community-dwelling older adults in Ethiopia.

**Methods:**

A community-based cross-sectional study was conducted among community-dwelling older adults of Gondar. Multi-stage random sampling technique was used across administrative areas. Six hundred and five households were selected proportionally using systematic random sampling technique. Physical measurement and face to face interview method were employed using a structured questionnaire for data collection. Data were analyzed descriptively and through uni- and multivariate logistic regression model.

**Results:**

One hundred and seventy (n = 170, 28.4%; 95% CI 24.7–32.1) community-dwelling older adults reported having experienced fall in the past 12 months. Sex (OR = 1.91, 95% CI: 1.24–2.95), low educational status (OR = 2.37, 95% CI: 1.19–4.74), uncomfortable home environment (OR = 2.02, 95% CI: 1.34, 3.04), having diagnosed medical condition (OR = 4.659, 95% CI: 1.20–18.02), and use of medication (OR = 5.57, 95% CI: 1.19–26.21) were significantly associated risk factors of self-reported fall in the past 12 months. Most outdoor falls are associated with females and participants aged below 66 years.

**Conclusion:**

In conclusion, more than 1/4th of the community-dwelling older adults experienced at least one episode of fall and about 60% of them reported recurrent falls. Identifying risk group and risk factors that could be modified so as to prevent falls in older adults deserves attention. Outdoor falls are usually attributable to modifiable environmental aspects and improvements in outdoor environment needed.

## Background

Falls are the leading cause of unintentional injuries and even premature death among community-dwelling older adults (CDOA) [1]. Falls are really a serious public health problem and are also the largest single cause of restricted activity and low life quality among older adults but usually a neglected public health problem in many societies, particularly in low and middle-income countries (LMICs) [2,3]. About 80% of disability resulting from unintentional injuries; which excludes traffic accidents in adults aged 50 years and over resulted from falls in the year 2010. In that year, years lived with disability due to fall in people aged 50 to 59 years was 66% in developing countries and 34% in high-income countries [4, 5]. Aging-related physical and mental changes along with other multifactorial variables increase the susceptibility and suffering of fall among older adults. Even in the absence of injury; a fall has potential consequences on quality of life due to fear of falling and, restricted activity to avoid falling again together with psychological changes like little confidence to get out [6,7]. World Health Organization (WHO) reports that the burden of non-intentional injuries are disproportionately higher in developing countries and older adults are at higher risk [8]. Reported results from the WHO Study on global AGEing and adult health (SAGE) from six countries including two sub-Saharan countries showed that two-thirds of all past-year injuries in older adults were fall-related and also suggested that data on falls among CDOA in LMICs is sparse [9]. The prevalence rate of falls among CDOA reported in the community-based studies done in Nigeria, Rwanda, South Africa, and Ghana range from 23% to 44% with a diverse definition for older adults across these studies [2]. Studies had reported a wide range of risk factors for falls in older adults like age gender, muscle weakness, gait disturbance, impaired balance, impaired vision, poor sleep pattern, depression, Parkinson’s, stroke, diabetes mellitus (DM), hypertension (HTN), dementia, and impaired cognition. Older adults living in LMICs encounter other risk factors like lack of awareness delayed health care, inadequate lighting, challenging outdoor environment, poor housing facilities, low education level, nutritional deficiency, and co-morbid [3,10,11]. In addition, the extensive variation found across studies among different countries and even within the same country clarifies the differential interaction of socio-economical, geographical and ethnic factors with the prevalence and risk factors of fall and the need for regional data [12,13]. Even though several literatures urge that the Governments in LMICs urgently require data and evidence to develop awareness, and integrate falls prevention into their policy and planning frameworks [2,14–17]; there is still a lack of data, on magnitude of falls among community-dwelling adults in most of the LMICs and till date there is no formal community based falls register and follow up data in Ethiopia as a whole. Hence, this study aims to determine the burden of self-reported past-year unintentional fall among CDOA of Gondar town in Ethiopia and identify the risk factors of fall.

## Material and method

### Study design and study setting

A community-based cross-sectional study was conducted from March to June 2018 in Gondar city, Northwest Ethiopia, situated in northern part of Ethiopia in Amhara National regional state and 747 km from the capital city Addis Ababa at 12° 45`north latitude and 37 °.45`east longitudes with an elevation of 2,706 meters (8,878 ft) above sea level and with usual challenges of mountain terrain. Based on the 2016 population estimates of Gondar city administration bureau Gondar had a total population of 335,000 with 3200/km^2^, with an estimated total household count of 53725 and 182000 (52%) women. Gondar town has 24 administrative areas (kebeles) of which 12 are classified as sub-cities or urban kebeles, 11 are rural kebeles and 1 special kebele. The estimate of Gondar resident adults aged 50 and above is not known. This study was conducted with a sample of community-dwelling older adults of age >50 years living in Gondar town, aged 50 years or older, resident in the selected kebele, ability to walk with or without walking aids, and to give informed consent for participation in the study were included.

### Sample size determination and sampling

The sample size was determined by using the single population proportion formula [18]. Considering the proportion of people to suffer fall as 23% from a previous study done in Rwanda [19], a design effect of 2 and expected 10% non-response rate, the final sample size was calculated to be 605 households (S1 File). A multistage random sampling technique was designed to reach the required sample size. Among the total of 24 kebeles in the town, only 12 sub-cities were considered for this study due to financial constraint. With the list of households secure from the local administrative bureau, 5 kebeles out of 12 sub-cities or urban kebeles were selected by using computer-generated random numbers from a census list of all kebeles. Households were allocated proportionally for each kebele. Systematic random sampling was used (with sampling interval K = 33) to select the households. In case of more than one older adults living in the selected household, a lottery method decided participation. Study variables and data collectionOperational definition: “Fall is an event that results in a person coming to rest inadvertently on the ground or floor or other lower level and should be considered as a recurrent event as soon as a subject reported at least two falls in the past 12-months period” [20]. This study used community-dwelling older adults aged 50 years and above on the day of interview living at home, able to walk independently or with a mobility aid and native residents of Gondar. Considering that most of the old persons in sub-Saharan Africa work outside the formal sector, and thus have no formal retirement at age 60, relatively lower life expectancy in Ethiopia [21] and smaller size of older populations in the study area. Hence, this study used 50 years of age and above as a general definition of older adults. Presences of medical conditions were assessed with questions asking the participants if they had been told by a physician that they had comorbid conditions. The participants were also questioned if their self-perceived current health status is better or same or worse compared with the previous year. Comfortable home environment is defined the one which has good lighting, safe passages or sideways, non-slippery even floor, space for the usage of mobility aids, convenient doorsteps, and in-door toilet room. A face to face interview method was employed using a structured questionnaire for data collection and seven data collectors preferably physiotherapist (3 senior and 4 junior physiotherapists) and those who were randomly selected from the registered list. The data collectors were intensively trained for two days on the background knowledge of the study, questionnaire, and physical measurements and calibration skills by the principal investigator. The data collectors introduced themselves and explained the purpose of this study to the participants. Informed consent was obtained from each participant and in case absence of participant or locked door situation in a selected household during the first visit; the same household was marked and re-visited only once after 24 hours and in case of absence again the immediate next house was approached. If a randomly selected participant was not willing to participate it was considered as non-response and the refusal reasons were noted. To ensure data quality, the questionnaire was first prepared in English and then translated from English to local language (Amharic) and finally back to English by language expertise to check for consistency. A pre-test was done (10% of sample size) in Medihanialem kebele, which was not included in the sample area. The principal investigator (BJ) checked for clarity, completeness, and cleaned the data each day and prior to analysis.

## Data analysis

Data were coded and entered into Epi Info software version 7.0 and IBM Statistical Package for Social Sciences (SPSS) version 24 for Windows for statistical analyses. Data entry with the original data was done by the data collectors and the principal investigator supervising each other to enhance correctness. In addition, the data was checked by two other researchers for completeness, accuracy, and clarity. Descriptive statistics (frequencies, percentages, means and standard deviations (SD) were used for all participant characteristics and associated factors of self-reported falls. With self-reported falls (categories: no versus yes) as the dependent variable, bivariate and multivariate binary logistic regression analyses were executed to examine the association with different independent variables. Independent variables included in the regression models were, age (categorized 50 65 middle-aged, 66 80 older and > 80 fragile), BMI (categorized underweight, normal weight, overweight and obese), educational status (categorized not educated, primary, secondary, Diploma and above), safety of home environment (categorized comfortable versus uncomfortable), self-perceived current level of mobility (categorized independent versus dependent), frequency of fall (categorized one, two, three, and many times), self-reported presence of medical conditions like hypertension, CVD, stroke, poor memory, visual impairment, poor urine control (categorized no versus yes), and poor sleeping (categorized no versus yes). Variables were inputted into the model using forced entry and categories were used as covariates for detailed analyses. Results were considered statistically significant when 95% confidence intervals not containing unity (equal to *p-*value <0.05) for interaction terms and main effects. Initially, bivariate analyses were conducted and independent variables that were found statistically significant were included in multivariate analysis. When clear subgroups seemed present in the data, significance testing (Pearson χ^2^) and, if appropriately sized subgroups per category remained, logistic regression was performed.

### Ethical approval and consent to participate

Ethical approval was secured from the Ethical Review Committee of the College of Medicine and Health Sciences, University of Gondar, Ethiopia (Reference number of ethical approval: SOM/004/7/09). Written consent was obtained from all participants prior to the participation in the study.

## Results

### Sample characteristics

A total of 605 households with a person aged ≥50 years old were visited. Of those, 599 (99%) older adults consented to participate and this is more than 100% of the power calculated sample size (n = 550) and the common reason given for non-response was no time or busy. Of the 599 participants, 327 (54.6%) were females and about 42% (n 252) of total participants self-reported to be non-workers or homemakers. The mean age of the participants was 61 years (SD 20 years) and their age ranged from 50 to 90 years. Among the participants, 43.6% and 27% had no formal education and primary education (< 8 years of schooling) respectively. Among the participants, 81% (489) reported being independently mobile without any difficulty. Only 3.3% lived alone, majority co-resided with their family members. Less than 19% were retired. More sample characteristics are presented in Table 1.

**Table 1:** Socio-demographic characteristics and distribution of fall among community-dwelling older adults of Gondar, Ethiopia 2018, (N = 599).

Variables	Sample totals		Fallers		Non-fallers	
	n	(%)	n	(%)	N	(%)
**All participants**	**599**	**(100%)**	**170**	**(28.4%)**	**429**	**(71.6%)**
**Sex**						
Female	327	(54.6)	105	(61.8)	222	(51.7)
Male	272	(45.4)	65	(38.2)	207	(48.3)
**Age (years)**						
50–65	440	(73.5)	114	(67.1)	326	(76)
66–80	147	(24.5)	51	(30)	96	(22.4)
>80	12	(2.0)	5	(2.9)	7	(1.6)
**Level of education**						
Non educated	261	(43.6)	66	(38.8)	195	(45.5)
Primary school	161	(26.9)	46	(27.1)	115	(26.8)
Secondary school	108	(18.0)	34	(20)	74	(17.2)
Degree	47	(7.8)	16	(9.4)	31	(7.2)
Post graduate and above	17	(2.8)	8	(4.7)	9	(2.1)
Diploma or technical	5	(0.8)	0	(0)	5	(1.2)
**BMI (kg/m**^**2**^**)**						
Under weight	84	(14.0)	26	(15.3)	58	(13.5)
Normal	403	(67.3)	110	(64.7)	293	(68.3)
Over weight	99	(16.5)	27	(15.9)	72	(16.8)
Obese	13	(2.2)	7	(4.1)	6	(1.4)
**Marital status**						
Never married	22	(3.7)	5	(2.9)	17	(4)
Currently married	282	(47.1)	01	(0.6)	281	(65.5)
Divorced/separated	76	(12.7)	34	(20)	42	(9.8)
Widowed	113	(18.9)	29	(17.1)	84	(19.6)
Cohabitating	6	(1.0)	1	(0.6)	5	(1.2)
**Occupation**						
Non workers/home makers	252	(42%)	82	(48.2)	170	(39.6)
Unskilled	122	(20.4)	33	(19.4)	89	(20.7)
Professional	78	(13.0)	24	(14.1)	54	(12.6)
Managerial	75	(12.5)	10	(5.9)	65	(15.2)
Retired	72	(12.0)	21	(12.4)	51	(11.9)
**Home environment**						
Comfortable to do activities	347	(57.9)	80	(47.1)	267	(63.2)
Slippery floor	48	(8.0)	15	(8.8)	33	(7.7)
Inadequate lighting	42	(7.0)	17	(10.0)	25	(5.8)
Poor housing facilities	162	(27.0)	58	(34.1)	104	(24.2)
**Current health status**						
Very good	88	(14.7)	15	(8.8)	73	(17.0)
Good	222	(37.1)	53	(31.2)	169	(39.4)
Average	235	(39.2)	79	(46.5)	156	(36.4)
Poor	54	(9.0)	23	(13.5)	31	(7.2)
**Health status one year before**						
Better	213	(35.6)	75	(44.1)	138	(32.2)
Same	316	(52.8)	77	(45.3)	239	(55.7)
Worse	70	(11.7)	18	(10.6)	52	(12.1)
**Current level of mobility**						
Independent	489	(81.6)	124	(72.9)	365	(85.1)
With difficulty	40	(6.7)	21	(12.4)	19	(4.4)
With walking aids	70	(11.7)	25	(14.7)	45	(10.5)
**Smoking**						
Non-smoker	548	(91.5)	158	(92.9)	390	(90.9)
Previous-smoker	24	(4.0)	5	(2.9)	19	(4.4)
Current smoker	27	(4.5)	7	(4.1)	20	(4.7)
**Alcohol consumption**						
Never	153	(25.5)	33	(19.4)	120	(28)
Previously	124	(20.7)	36	(21.2)	88	(20.5)
Current	322	(53.8)	101	(59.4)	221	(51.5)
**Khat chewing**						
Never	557	(93.0)	160	(94.1)	397	(92.5)
Previously	16	(2.7)	0	(0)	16	(3.7)
Current	26	(4.3)	10	(5.9)	16	(3.7)

About 9% of the participant’s self-reported poor current health and 35.6% perceived their current health to be worse than a year ago. Almost half (n = 318) of the participants reported being diagnosed to have at least one comorbid. The median for comorbid conditions was one (range 0–9) and median for oral medications was one (range 0–12). Medical conditions commonly reported were visual problems (31.7%). hypertension (29.7%), foot disorders (19.4%), diabetes (11.2%), lung diseases (9%) and cardiovascular diseases (8%).

### Falls and distribution

**Fig 1:**
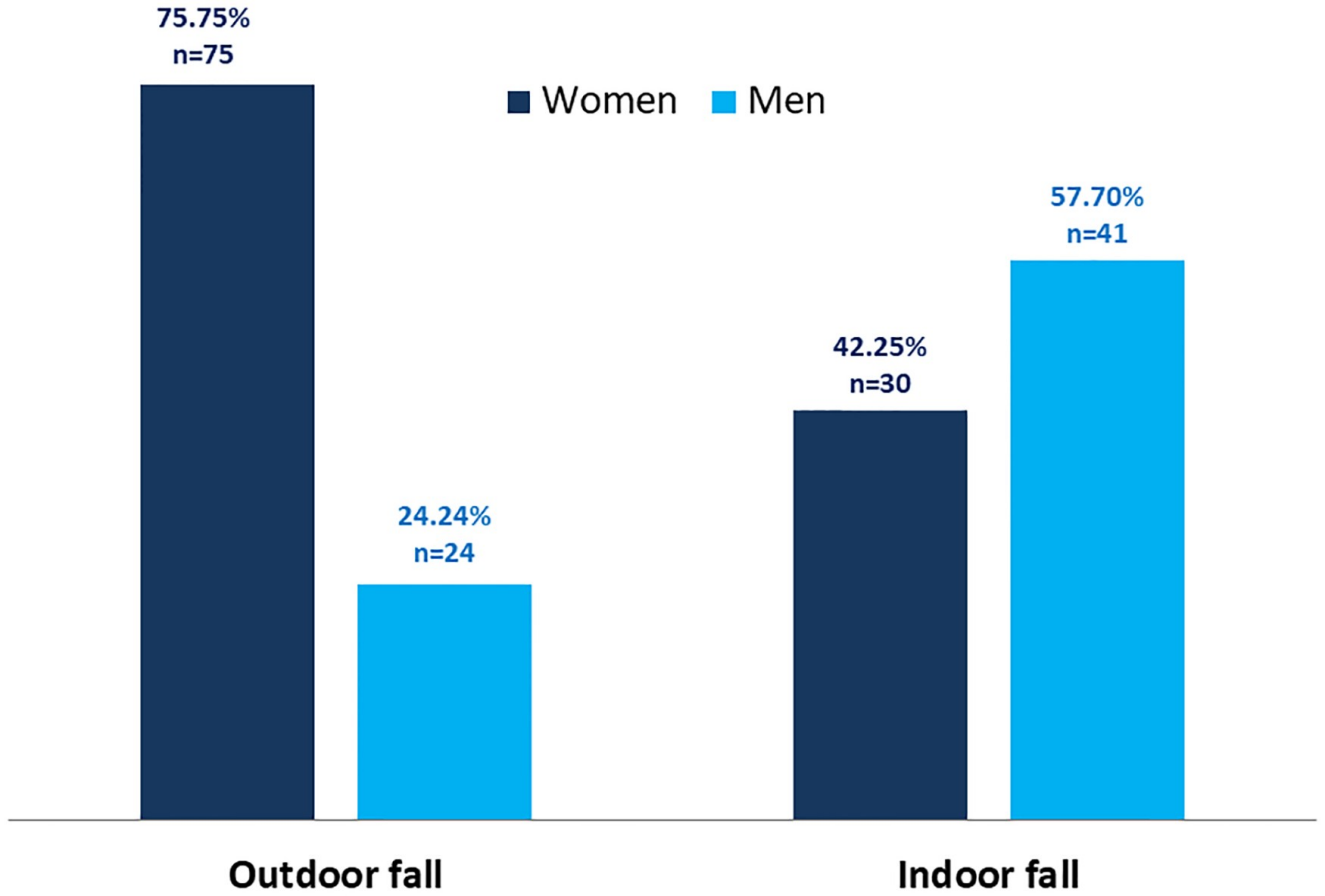
Frequency of indoor and outdoor fall among community-dwelling older adults, Gondar, Ethiopia; men versus women, Ethiopia.

One hundred and seventy (n = 170, 28.4%; 95% CI 24.7–32.1) CDOA reported to have experienced falls in the past 12 months. Among those who reported previous experience of falling, majority (60.5%) of them experienced recurrent falls. A statistically significant difference was observed in the prevalence rate of fall between genders (male 24.1% versus female 32.2%; χ^2^ (1, *n* = 599) = 18.1, p<0.01, *phi =* 0.19). A total of 351 falls were reported by the (n = 170) fallers, an average of 2.1 falls per faller and 2.74 falls per recurrent fallers. Among recurrent fallers, women (65%) reported more recurrent falls than men. Of the 103 participants who reported recurrent falls, 65% were women. Most falls among men occurred indoors (63.1% versus 28.9% for men and women, respectively, p<0.001); while older women reported more of outdoor falls (Fig 1). Falls in participants aged 65 years and below occurred mainly outdoors 53.5%; while those aged above 65 years falls occurred mainly indoors 81.2% than outdoor.

Frequency distribution of self-reported comorbid among fallers as diagnosed by physicians were; vision problems (37%), hypertension (37.6%), foot problems (28.2%), diabetes (17.1%), cardiovascular conditions (14.1%), and poor memory (7.6%). While17.6%, 22.9% and 9.4% fallers self-reported to have poor urine control, poor sleeping, and impaired hearing respectively Table 2.

**Table 2:** Self-reported as physician-diagnosed and self-reported clinical characteristics of participants, Gondar, Ethiopia 2018, (N = 599).

	Sample totals		Fallers		Non-fallers	
	N	(%)	n	(%)	n	(%)
**Medical conditions**						
None	281	(46.9)	50	(30)	230	(53.6)
At least one	318	(53.1)	120	(70)	199	(46.4)
**Hypertension**						
No	421	(70.3)	64	(37.6)	114	(26.6)
Yes	178	(29.7)	106	(62.4)	315	(73.4)
**Diabetes mellitus**						
No	532	(88.8)	29	(17.1)	38	(8.9)
Yes	67	(11.2)	141	(82.9)	391	(91.1)
**Stroke**	0	(0.0)				
No	583	(97.3)	7	(4.1)	9	(2.1)
Yes	16	(2.7)	163	(95.9)	420	(97.9)
**Cardiovascular disease**						
No	551	(92.0)	24	(14.1)	24	(5.6)
Yes	48	(8.0)	146	(85.9)	405	(94.4)
**Chronic lung disease**						
No	545	(91.0)	151	(88.8)	394	(91.8)
Yes	54	(9.0)	19	(11.2)	35	(8.2)
**Foot problems**						
No	483	(80.6)	122	(71.8)	361	(84.1)
Yes	116	(19.4)	48	(28.2)	68	(15.9)
**Dizziness**						
No	563	(94.0)	156	(91.8)	407	(94.9)
Yes	36	(6.0)	14	(8.2)	22	(5.1)
**Parkinson**	0	(0.0)				
No	597	(99.7)	168	(98.8)	429	(100)
Yes	2	(0.3)	2	(1.2)	0	(0)
**Vertigo**						
No	556	(92.8)	156	(91.8)	400	(93.2)
Yes	43	(7.2)	14	(8.2)	29	(6.8)
**Poor memory**						
No	571	(95.3)	157	(92.4)	414	(96.5)
Yes	28	(4.7)	13	(7.6)	15	(3.5)
**Depression**						
No	587	(98.0)	166	(97.6)	421	(98.1)
Yes	12	(2.0)	4	(2.4)	8	(1.9)
**Cancer**						
No	596	(99.5)	170	(100)	426	(99.3)
Yes	3	(0.5)	0	(0)	3	(0.7)
**Cognitive problem**						
No	599	(100.0)	170	(100)	429	(100)
Yes	0	(0.0)	0	(0.0)	0(0.0)	
**Number of comorbid (median, (IQR)**	1	(0–2)	1,	(0–2)	1	(0–1)
**Total drug intake (median, IQR)**	1	(0–3)	3,	(0–4.3)	1	(0–3)
**Poor urine control**						
Yes	57	(9.5)	30	(17.6)	27	(6.3)
No	542	(90.5)	140	(82.4)	402	(93.7)
**Poor sleeping**						
No	490	(81.8)	131	(77.1)	359	(83.7)
Yes	109	(18.2)	39	(22.9)	70	(16.3)
**Vision problem**						
Yes	190	(31.7)	70	(37)	120	
a) Near: poor	89	(14.9)	37	(21.8)	52	(12.1)
b) Far: poor	101	(16.9)	33	(19.4)	68	(15.9)
No	409	(68.3)	100	(58.8)	309	(72)
*Vision*						
Near: poor	89	(14.9)	37	(21.8)	52	(12.1)
Far: poor	101	(16.9)	33	(19.4)	68	(15.9)
**Impaired Hearing**						
Yes	42	(7.0)	16	(9.4)	26	(6.1)
No	557	(93.0)	154	(90.6)	403	(93.9)

### Associated factors of falls

Gender was significantly different among fallers (χ^2^ (1, n = 599) = 492.8, p< 0.05, phi = 0.91 with most females (n = 105, 61.8% versus 38.2%) reporting fall in the past 12 months. And, female gender was also found to be associated with recurrent falls (χ^2^ (1, n = 103) = 54.5, p< 0.01, phi = 0.96 (n = 67, 65% versus 35%) compared to males. Of those who reported of being independent, which constitutes a subgroup in which a greater prevalence of fall in past 12 months was found (n = 124, 72.9% versus 12.4% and 14.7%) and significantly different (χ^2^ (2, n = 599) = 154.9, p< 0.001, phi = 0.16 from the other sub groups. self-reported fall prevalence Table 3.

**Table 3:** Fall-related characteristics of (CDOA) fallers of Gondar, Ethiopia 2018 (n-170).

Fall related variables	Female	Male	Total	p-value
	n (%)	n (%)	n (%)	
**No of falls in the previous year**				
One	38 (36.2)	29 (44.6)	67 (39.4)	
Two	34 (32.4)	24 (36.9)	58 (34.1)	
Three	19 (18.1)	9 (13.8)	28 (16.5)	
Many times	14 (13.3)	3 (4.6)	17 (10.0)	0.216
**Place of fall**				
Indoor	30 (28.6)	41 (63.1)	71 (41.8)	
Outdoor	75 (71.4)	24 (36.9)	99 (58.2)	0.000*
**Mechanism of the fall**				
Tripped	32 (30.5)	20 (30.8)	52 (30.6)	
Slipped	38 (36.2)	19 (29.2)	57 (33.5)	
I lost my balance	5 (4.8)	5 (7.7)	10 (5.9)	
My Legs gave away	15 (14.3)	10 (15.4)	25 (14.7)	
Fainted	3 (2.9)	4 (6.2)	7 (4.1)	
Felt dizzy	5 (4.8)	6 (9.2)	11 (6.5)	
Not sure	7 (6.)	1 (1.5)	8 (4.7)	0.453
**Seeking medical attention after fall**				
Yes	81 (77.1)	52 (80)	133(78.2)	
No	24 (22.9)	13 (20)	37 (21.8)	0.661

*Denotes significant difference, Chi-square test was used for categorical variables

Among the fallers majority of them had one or more comorbid (χ^2^ (1, n = 170) = 272), and presence of comorbid is significantly associated with a tendency to fall p< 0.001, phi = 0.21 (n = 119, 70% versus 30%). Other variables that were associated with the previous 12 months fall were drug intake, HTN, DM, poor memory, poor urine control, and CVD. Place of fall was significantly different for men and women. Other variables potentially related to fall showed no variance between genders or between fallers and non-fallers.

### Regression analysis

Prior to analysis variables potentially related to fall were identified for bivariate logistic regression model: age, sex, BMI, educational status, occupation, marital status, family size, home environment, self-reported health status, current level of mobility, physical activity, medical condition, use of medication, HTN, Stroke, DM, CVD, Foot problem, vision problem, impaired hearing, poor memory, poor urine control, poor sleeping, alcohol consumption, place of fall, and mechanism of fall. Of those, the remaining variables were significant and were evaluated using multivariate model (Table 3). Sex (OR = 1.91, 95% CI: 1.24–2.95), low educational status (OR = 2.37, 95% CI: 1.19–4.74), uncomfortable home environment (OR = 2.02, 95% CI: 1.34, 3.04), having diagnosed medical condition (OR = 4.659, 95% CI: 1.20–18.02), and use of medication (OR = 5.57, 95% CI: 1.19–26.21) were significantly associated with self-reported fall in the past 12 months (Table 4). Among medical conditions, poor memory (OR = 5.57. CI: 1.19, 10.21) and poor urine control (OR = 3.28. CI: 1.61, 6.70) were more likely associated with falls. We found a non-significant interaction effect when placed potential variables together in the model and no difference in slopes in the interaction effects plot.

**Table 4:** Associated factors of falls among community-dwelling older adults in Gondar, North-West Ethiopia 2018 (N = 599).

Variable	Fallers	Non-fallers	COR (95%) CI	AOR (95%) CI	p
**Sex**					
Male	65	207	1.506(1.05, 2.16)	1.48(0.63,3.46)	0.363
Female	105	222	1(ref)	1(ref)	
**Age**					
50-65	114	326	1(ref)	1(ref)	
66-80	51	96	1.52(1.017, 2.27)	0.85(0.49, 1.4)	0.57
>80	5	7	2.04(0.64, 6.5)	0.94(0.23, 3.8)	0.94
**Educational status**					
No education	66	195	1(ref)	1(ref)	
Primary	46	115	1.18(1.06, 1.83)	**1.45(1.81, 2.23)**	0.004*
Secondary	34	74	1.35(1.83, 2.22)	**2.25(1.27, 3.92)**	0.034*
Diploma & above	24	45	1.58(1.89, 2.78)	**2.48(1.28, 4.73)**	0.048*
**Family size**					
Living alone	9	11	1(ref)	1(ref)	
Living with someone	161	418	0.47(0.19, 1.16)	0.83(0.12, 5.57)	0.85
**Home environment**					
Comfortable	80	267	0.54(0.38,0.77)	**0.44(0.21, 0.95)**	0.036*
Uncomfortable	90	162	1(ref)	1(ref)	
**Current level of mobility**					
Independent	126	366	1(ref)	1(ref)	0.47
Dependent	44	63	2.03(1.31,3.13)	0.59(0.14, 2.45)	
**Current level of mobility**					
Independent	365	124	1(ref)	1(ref)	
With difficulty	45	25	3.25(1.69, 6.25)	**2.59(1.24, 5.41)**	**0.011**
With walking aids	19	21	1.64(1.06, 2.77)	1.23(0.64, 2.35)	0.539
**Medical condition**					
Yes	120	199	2.77(1.89, 4.06)	**4.66(1.20, 18.02)**	0.026*
No	50	230	1(ref)	1(ref)	
**Number of medication/day**					
No medication	56	235	1(ref)	1(ref)	
1-3 drugs	71	131	2.27(1.51, 3.43)	1.27(0.63, 2.5)	0.49
More than 3	43	63	2.86(1.76, 4.65)	1.11(0.40.3.1)	0.83
**HTN**					
Yes	55	103	1.51(1.02, 2.23)	2.85(0.93, 8.73)	0.066
No	115	326	1(ref)	1(ref)	
**Stroke**					
Yes	7	9	2.04(0.73,5.47)	0.51(0.09, 3.03)	0.46
No	163	420	1(ref)	1(ref)	
**DM**					
Yes	29	38	2.11(1.26, 3.56)	2.55(0.68, 9.61)	0.167
No	141	391	1(ref)	1(ref)	
**CVD**					
Yes	24	24	2.77(1.53, 5.04)	0.44(0.12, 1.57)	0.205
No	146	405	1(ref)	1(ref)	
**Foot problem**					
Yes	48	68	2.09(1.37, 3.19)	1.28(0.47, 3.46)	0.632
No	122	361	1(ref)	1(ref)	
**Vision problem**					
Yes	70	120	1.80(1.24, 2.61)	0.62(0.26, 1.47)	0.277
No	100	309	1(ref)	1(ref)	
**Poor memory**					
Yes	13	15	1.03(0.36, 2.94)	**5.57(1.19, 10.21)**	0.030*
No	157	414	1(ref)	1(ref)	
**Poor urine control**					
Yes	30	27	3.2(1.8, 5.5)	**3.28(1.61, 6.70)**	0.001*
No	140	420	1(ref)	1(ref)	
**Alcohol consumption**					
Never	33	120	1(ref)	1(ref)	
Previously	36	88	1.66(1.06, 2.61)	1.50(0.57, 3.97)	0.414
Current	101	221	1.117(0.71, 1.76)	1.56(0.58, 4.15)	0.378

* Denotes significant association of characteristics with fall in multivariate model,

AOR- Adjusted odds ratio, CI -Confidence Interval, COR-Crude odds ratio.

Note: R^2^ = 0.142 (Cox & Snell), 0.204 (Nagelkerke). Model X^2^ (3, n = 599) = 92.01, p<0.001. Correctly predicted: 76.3%.

## Discussion

To our knowledge, this is a preliminary community-based study in the country to concurrently estimate the magnitude of fall and describe the relationships between fall and risk factors in community-dwelling older adults in Ethiopia. The results of the present study indicate that 28.4% of community-dwelling older adults aged 50 years or older sustained fall each year in Ethiopia. Among them, about 39.1% reported having occasional falls and 60.9% recurrent falls. The finding of the present study is within the global annual fall rate which is between 6% and 35% [8,22,23]. The reported rate of fall appears to vary among countries; in South-East Asia region, in China, and Japan, 6–31% and 20% respectively [24], Latin American and Caribbean region, the fall rates reported were 21.6% and 34% respectively [8]. In the Sub-Saharan region; the reported fall rate of Nigerian older adults is 23% and 44% in Ghana [2,25]. The definition for older adult widely varied among the studies and more so the fall rate and factors associated among the older population in Ethiopia is not reported elsewhere. The result of this study is higher when compared to the studies done in Malaysia 4.1%, Japan 15.9%, Hong Kong 19.3%, and twelve European countries 7.9–16.2% [22,24–28]. The possible explanations could be reflecting medical conditions, geographical challenges of mountain terrain of the study area, and study population differences compared to other countries. In this study, more than half of the recruited participants (53.3%) reported being diagnosed with one or more medical conditions. Frail, elderly people with more than one chronic illness experience higher rates of falls than active healthy older people [5,15]. The prevalence reported in this study is much lower than self-reported falls of Mexican and Jamaican community-dwelling older adults 46.5% and 51.5% [29,30]. This could possibly be due to the lower mean age of participants in this study. Around 3/4^th^ of the participants (73.5%) in this study were aged 50–65 years. In Mexico fall was assessed for the previous two years, all participants’ were aged 60 years and above, and the mean age of all those who experienced a fall was 71.4 years. Different epidemiological studies describe multi-factorial risks for fall among elders [2,5,28]. According to WHO, there are four dimensions; biological, behavioral, environmental and socioeconomic factors [8]. In agreement with previous studies [4,10,26], this study also found that women were more likely than men to have double folded magnitude of fall and recurrent falls. In this study female gender was also one point nine times higher risk of fall as compared to the male which is likewise studies in Mexico, Rwanda, and Indonesia [9,29,31]. The reasons might be gender-specific physiologic changes like sarcopenia, decreased strength, loss of bone density, and social reasons leading to more falls and related injuries [32]. Similarly, in Ethiopia females are involved in over half of the farm activities, bear most of the responsibilities in the household, and income generation [33,34]. Furthermore, women being frailer, live longer than men, and involved in heavy household works partly explains their susceptibility to falling and more so indoor falls [4,35]. The finding of this study shows that uneducated community-dwelling elders are twice more likely to fall than their educated counterpart. Lower education level as a possible predictor of fall among elders is reported by studies elsewhere [2,5,36]. One possible reason might be low education status and/or uneducated elders may lack a proper understanding of aging consequences and relative life adjustment needed. It is known that drastic change in educational attainment leads to greater improvement in the elder’s health [37]. Moreover, fall data among uneducated older are scant in studies from developed countries [26–28] unlike in this study nearly 40% of the participants are uneducated. CDOA those who reported living in an uncomfortable environment have two times more risk of falling than those reported living in comfortable environments. With older adults of this study reported spending little time outdoors compared to indoors, it is surprising that we found the frequency of outdoor falls were higher compared with indoor falls among CDOA in the study area which possibly explains the challenges of mountain terrain. Similar findings were reported by studies done in California and Norway [22,37]. It is particularly striking because leisure-time physical activity was not associated with fall in this study. Another unexpected finding in this study is women reported more outdoor falls and the most likely reason could be women engaging in more outdoor activities to fulfill domestic needs of households. It is understandable that outdoor falls are most probably caused by environmental challenges along with intrinsic risk factors. The study place, Gondar is located 2133 meters above sea level with geographical challenges for access to domestic needs, facilities and walking [38]. Furthermore, in developing countries like Ethiopia environments pose challenges like uneven roads, lack of designed walkways, sidewalks, lack of street lights, more stairs or tripping, and slipping hazards. About 70% of CDOA in this study self-reported of being diagnosed with at least one comorbid, the presence of comorbid contribute to an elevated prevalence of falls and five folds higher risks of falls than of healthy elders. This association is consistent with the studies in South Africa and Egypt [39,40]. The possible explanations could be various medical conditions like vision problem, foot problems cardiovascular issues and motor performance deficits for older adults appear due to dysfunction and reduced coordination of the central and peripheral nervous systems and the neuromuscular system are implicated to increase an individual’s risk of falling [39,41]. Though unlike elsewhere, we found no association with fall and self-reported dizziness and vertigo which is difficult to explain. The number of medications taken, unlike in other studies was significantly associated risk factors with falls in this study [22,42,43]. Elders those using medications for their conditions are twice at the risk of falling compared to non-users. This finding is similar to few studies in high-income countries, South Africa, and Egypt [27,39,40]. An association between use of medications and fall risk may depend on the prescribing habits of health practitioners (type of medications commonly taken by the subjects), the site of action of the drug and adverse effects of the drugs. There is increasing evidence that poly-pharmacy may lead to falls as a result of adverse reactions to one or more medications, detrimental drug interaction, and/or incorrect use [42,43]. The variety of classes of medications prescribed, which results in insufficient numbers of individuals taking a particular class of drug, hampers meaningful contribution to the analyses of the individual drug classes [19,35,44, 45]. To benefit future researches and interpreting the present result with caution the limitations must be mentioned. First, because of the cross-sectional nature of this study, we cannot determine the causal effects. Moreover, self-reports of socio-demographic, health characteristics and medical diagnoses by older adults may be a source of recall bias. Second, data on specific medications, nutritional status, fall-related risk-taking behavior, and housing conditions were not investigated. Despite these limitations, this study is a preliminary attempt in this country to provide a well-powered insight and estimate the prevalence of fall and to examine characteristics associated among CDOA in Ethiopia. In addition in this study, the definition of older adults being aged 50 years and above is according to the recommendations of World Confederation for Physical Therapy (WCPT) and WHO Older adult Health and Ageing in Africa project. We strongly feel that these data will more accurately determine the fall burden of the older population in the study region and would fully inform policy makers and program planners.

## Conclusion and recommendation

In conclusion, more than 1/4^th^ of the CDOA experienced at least one episode of fall and about 60% of them reported recurrent falls. Factors like comorbid, medication, self-reported poor memory and poor urine control explained fall among CDOA partly. Additional factors that may help explain fall in CDOA should be explored in the future. In the meantime, town authorities are recommended to maintain preventable environmental risk factors related to outdoor falls. Caretakers, older adults, and family members should be made aware of the possible risk factors and modifications needed to avoid fall.

## Supporting information

S1 File Sample size calculation and flow chart of sampling procedure. (PDF)

S2 File STROBE Statement—Checklist of items for fall among community dwelling older adults in Ethiopia. (DOCX)

## Abbreviations

AOR: Adjusted odds ratio
BMI: Body Mass Index
CDOA: Community-dwelling older adults
CI: Confidence interval
COR: crude odds ratio
CSA: central statistics Agency
CVD: Cardiovascular disease
DM: Diabetic Mellitus
HTN: hypertension
LMICs: Low Middle income countries
OR: Odds Ratio
SAGE: Study on global AGEing and adult health
WCPT: World Conference for Physical Therapy
WHO: World Health Organization

## References

[1] Yoo IY. Recurrent falls among community-dwelling older Koreans: prevalence and multivariate risk factors. J Gerontol Nurs. 2011;37(9):28–40. 10.3928/00989134-20110503-01 21634315

[2] Williams JS, Kowal P, Hestekin H, O’Driscoll T, Peltzer K, Yawson A, et al Prevalence, risk factors and disability associated with fall-related injury in older adults in low-and middle-incomecountries: results from the WHO Study on global AGEing and adult health (SAGE). BMC Med. 2015;13(1):147. 26099794 10.1186/s12916-015-0390-8 PMC4495610

[3] Coutinho ES, Fletcher A, Bloch KV, Rodrigues LC. Risk factors for falls with severe fracture in elderly people living in a middle-income country: a case control study. BMC Geriatr. 2008;8(1):21. 18727832 10.1186/1471-2318-8-21 PMC2532993

[4] Kowal KP, Chatterji S. Measuring prevalence and risk factors for fall-related injury in older adults in low-and middle-income countries: results from the WHO Study on Global AGEing and Adult Health (SAGE). WHO Working Paper; 2013.

[5] Ambrose AF, Paul G, Hausdorff JM. Risk factors for falls among older adults: a review of the literature. Maturitas. 2013;75(1):51–61. 10.1016/j.maturitas.2013.02.009 23523272

[6] Rubenstein LZ, Josephson KR. Falls and their prevention in elderly people: what does the evidence show? Med Clin. 2006;90(5):807–24. 10.1016/j.mcna.2006.05.013 16962843

[7] Deshpande N, Metter EJ, Bandinelli S, Lauretani F, Windham BG, Ferrucci L. Psychological, physical and sensory correlates of fear of falling and consequent activity restriction in the elderly: The InCHIANTI Study. Am J Phys Med Rehabil Acad Physiatr. 2008;87(5):354. 10.1097/PHM.0b013e31815e6e9b PMC2495025 18174852

[8] World Health Organization. Ageing; Life Course Unit. WHO global report on falls prevention in older age. World Health Organ. 2008;

[9] Kalula SZ, Ferreira M, Swingler GH, Badri M, Sayer AA. Methodological challenges in a study on falls in an older population of Cape Town, South Africa. Afr Health Sci. 2017;17(3):912–22. 10.4314/ahs.v17i3.35 29085420 PMC5656198

[10] Chang VC, Do MT. Risk factors for falls among seniors: implications of gender. Am J Epidemiol. 2015;181(7):521–31. 10.1093/aje/kwu268 25700887

[11] Jember G, Melsew YA, Fisseha B, Sany K, Gelaw AY, Janakiraman B. Peripheral Sensory Neuropathy and associated factors among adult diabetes mellitus patients in Bahr Dar, Ethiopia. J Diabetes Metab Disord. 2017;16(1):16. 28396852 10.1186/s40200-017-0295-5 PMC5381058

[12] Denkinger MD, Lukas A, Nikolaus T, Hauer K. Factors associated with fear of falling and associated activity restriction in community-dwelling older adults: a systematic review. Am J Geriatr Psychiatry. 2015;23(1):72–86. 10.1016/j.jagp.2014.03.002 24745560

[13] Stubbs B, Binnekade T, Eggermont L, Sepehry AA, Patchay S, Schofield P. Pain and the risk for falls in community-dwelling older adults: systematic review and meta-analysis. Arch Phys Med Rehabil. 2014;95(1):175–87. 10.1016/j.apmr.2013.08.241 24036161

[14] Chandran A, Hyder AA, Peek-Asa C. The Global Burden of Unintentional Injuries and an Agenda for Progress. Epidemiol Rev. 2010 4 1;32(1):110–20. 20570956 10.1093/epirev/mxq009 PMC2912603

[15] de Ramirez SS, Hyder AA, Herbert HK, Stevens K. Unintentional Injuries: Magnitude, Prevention, and Control. Annu Rev Public Health. 2012 3 19;33(1):175–91. 22224893 10.1146/annurev-publhealth-031811-124558

[16] Norton R, Kobusingye O. Injuries. N Engl J Med. 2013 5 1;368(18):1723–30. 10.1056/NEJMra1109343 23635052

[17] Unintentional injuries: magnitude, prevention, and control. 10.1146/annurev-publhealth-031811-124558 22224893

[18] Kasiulevičius V, Šapoka V, Filipavičiūtė R. Sample size calculation in epidemiological studies. Gerontologija. 2006;7(4):225–31.

[19] Ntagungira EK. Epidemiology of and risk factors for falls among the community-dwelling elderly people in selected districts of Umutara Province, Republic of Rwanda. 2005;

[20] BeauchetO, FantinoB, AllaliG, MuirS, Montero-OdassoM, AnnweilerC. Timed Up and Go test and risk of falls in older adults: a systematic review. J Nutr Health Aging. 2011;15(10):933–8. 2215978510.1007/s12603-011-0062-0

[21] WHO | Proposed working definition of an older person in Africa for the MDS Project [Internet]. WHO. [cited 2019 Jul 10]. http://www.who.int/healthinfo/survey/ageingdefnolder/en/

[22] Sousa LMM, Marques-Vieira CMA, de Caldevilla MNGN, Henriques CMAD, Severino SSP, Caldeira SMA. Risk for falls among community-dwelling older people: systematic literature review. Rev Gaucha Enferm. 2016;37(4). 10.1590/1983-1447.2016.04.55030 28273251

[23] Bergland A. Fall risk factors in community-dwelling elderly people. 2012;

[24] Hua F, Yoshida S, Junling G, Hui P. Falls prevention in older age in Western Pacific Asia Region. WHO Backgr Pap Glob Rep Falls Older Pers. 2007.

[25] Bekibele C, Gureje O. Fall incidence in a population of elderly persons in Nigeria. Gerontology. 2010;56(3):278–83. 10.1159/000236327 19738364 PMC2862232

[26] Yeong U, Tan S, Yap J, Choo W. Prevalence of falls among community-dwelling elderly and its associated factors: A cross-sectional study in Perak, Malaysia. Malays Fam Physician Off J Acad Fam Physicians Malays. 2016;11(1):7. PMC5405326 28461842

[27] Kitayuguchi J, Kamada M, Okada S, Kamioka H, Mutoh Y. Association between musculoskeletal pain and trips or falls in rural J apanese community‐dwelling older adults: A cross‐sectional study. Geriatr Gerontol Int. 2015;15(1):54–64. 10.1111/ggi.12228 24418209

[28] Franse CB, Rietjens JA, Burdorf A, van Grieken A, Korfage IJ, van der Heide A, et al A prospective study on the variation in falling and fall risk among community-dwelling older citizens in 12 European countries. BMJ Open. 2017;7(6):e015827 10.1136/bmjopen-2017-015827 28667220 PMC5726118

[29] Agudelo-Botero M, Giraldo-Rodríguez L, Murillo-González JC, Mino-León D, Cruz-Arenas E. Factors associated with occasional and recurrent falls in Mexican community-dwelling older people. PloS One. 2018;13(2):e0192926 10.1371/journal.pone.0192926 29462159 PMC5819783

[30] Mitchell-Fearon K, James K, Waldron N, Holder-Nevins D, Willie-Tyndale D, Laws H, et al Falls Among Community-Dwelling Older Adults in Jamaica. SAGE Open. 2014;4(4):2158244014564351.

[31] Pengpid S, Peltzer K. Prevalence and Risk Factors Associated with Injurious Falls among Community-Dwelling Older Adults in Indonesia. Curr Gerontol Geriatr Res. 2018;2018. 10.1155/2018/5964305 PMC6008814 29971097

[32] White AM, Tooth LR, Peeters GG. Fall Risk Factors in Mid-Age Women: The Australian Longitudinal Study on Women’s Health. Am J Prev Med. 2018;54(1):51–63. 10.1016/j.amepre.2017.10.009 29254554

[33] Tegegne M. An assessment on the role of women in agriculture in Southern Nation Nationality People’s Region: The case of Halaba Special Woreda, Ethiopia. 2012.

[34] Koolwal G, Van de Walle D. Access to water, women’s work, and child outcomes. Econ Dev Cult Change. 2013;61(2):369–405.

[35] Gillespie LD, Robertson MC, Gillespie WJ, Sherrington C, Gates S, Clemson LM, et al Interventions for preventing falls in older people living in the community. Cochrane Database Syst Rev. 2012;(9). 10.1002/14651858.CD007146.pub3 PMC8095069 22972103

[36] World Health Organization. WHO global report on falls prevention in older age. 2007 World Health Organ 2015;1–7.

[37] Kye B, Arenas E, Teruel G, Rubalcava L. Education, elderly health, and differential population aging in South Korea: A demographic approach. Demogr Res. 2014;30:753–94.

[38] Li W, Keegan TH, Sternfeld B, Sidney S, Quesenberry CP Jr, Kelsey JL. Outdoor falls among middle-aged and older adults: a neglected public health problem. Am J Public Health. 2006;96(7):1192–200. 10.2105/AJPH.2005.083055 16735616 PMC1483851

[39] Okwaraji YB, Webb EL, Edmond KM. Barriers in physical access to maternal health services in rural Ethiopia. BMC Health Serv Res. 2015;15(1):493. 26537884 10.1186/s12913-015-1161-0 PMC4634737

[40] Kalula SZ, Ferreira M, Swingler GH, Badri M. Risk factors for falls in older adults in a South African Urban Community. BMC Geriatr. 2016;16(1):51. 26912129 10.1186/s12877-016-0212-7 PMC4766747

[41] Kamel MH, Abdulmajeed AA, Ismail SE-S. Risk factors of falls among elderly living in Urban Suez-Egypt. Pan Afr Med J. 2013;14(1). 10.11604/pamj.2013.14.26.1609 PMC3597910 23504298

[42] Ambrose AF, Paul G, Hausdorff JM. Risk factors for falls among older adults: a review of the literature. Maturitas. 2013;75(1):51–61. 10.1016/j.maturitas.2013.02.009 23523272

[43] De Jong MR, Van der Elst M, Hartholt KA. Drug-related falls in older patients: implicated drugs, consequences, and possible prevention strategies. Ther Adv Drug Saf. 2013;4(4):147–54. 10.1177/2042098613486829 25114778 PMC4125318

[44] Richardson K, Bennett K, Kenny RA. Polypharmacy including falls risk-increasing medications and subsequent falls in community-dwelling middle-aged and older adults. Age Ageing. 2014;44(1):90–6. 10.1093/ageing/afu141 25313240

[45] Bruce J, Lall R, Withers EJ, Finnegan S, Underwood M, Hulme C, et al A cluster randomised controlled trial of advice, exercise or multifactorial assessment to prevent falls and fractures in community-dwelling older adults: protocol for the prevention of falls injury trial (PreFIT). BMJ Open. 2016;6(1):e009362 10.1136/bmjopen-2015-009362 26781504 PMC4735205

