# Supplementary material for: Falls among community-dwelling older adults in Ethiopia; A preliminary cross-sectional study
Source: PLoS One. 2019 Sep 10;14(9):e0221875. doi: 10.1371/journal.pone.0221875 (PMC6736232; doi:10.1371/journal.pone.0221875)

APPENDIX I

Sample size determination and sampling procedure

Sample size was determined using the formula for single population proportion by considering Prevalence of [Expected frequency] 23.3% (1)

$$n = g * \frac{Z^2 \alpha/2 p (1-p)}{w^2} = 2 * \frac{(1.96)^2 (0.23)(1-0.23)}{(0.05)^2} = 550 \text{ households [power calculated sample]}$$

- α = (level of significance) = 5%
- Z = value of z statistic at 95% confidence level
- w = margin of error allowed = 5%
- P = (Expected people to have experience at least one fall in the previous one year)
- q = 1 – p = 77
- g = design effect
- n = sample size

Considering a contingency of 10% was added, the final sample size (households) derived was:

605 households

Reference

1. Ntagungira EK. Epidemiology of and risk factors for falls among the community-dwelling elderly people in selected districts of Umutara Province, Republic of Rwanda. 2005;

APPENDIX 2

A flow chart of sampling procedure followed in households of 12 sub cities of Gondar town included in the study, 2018

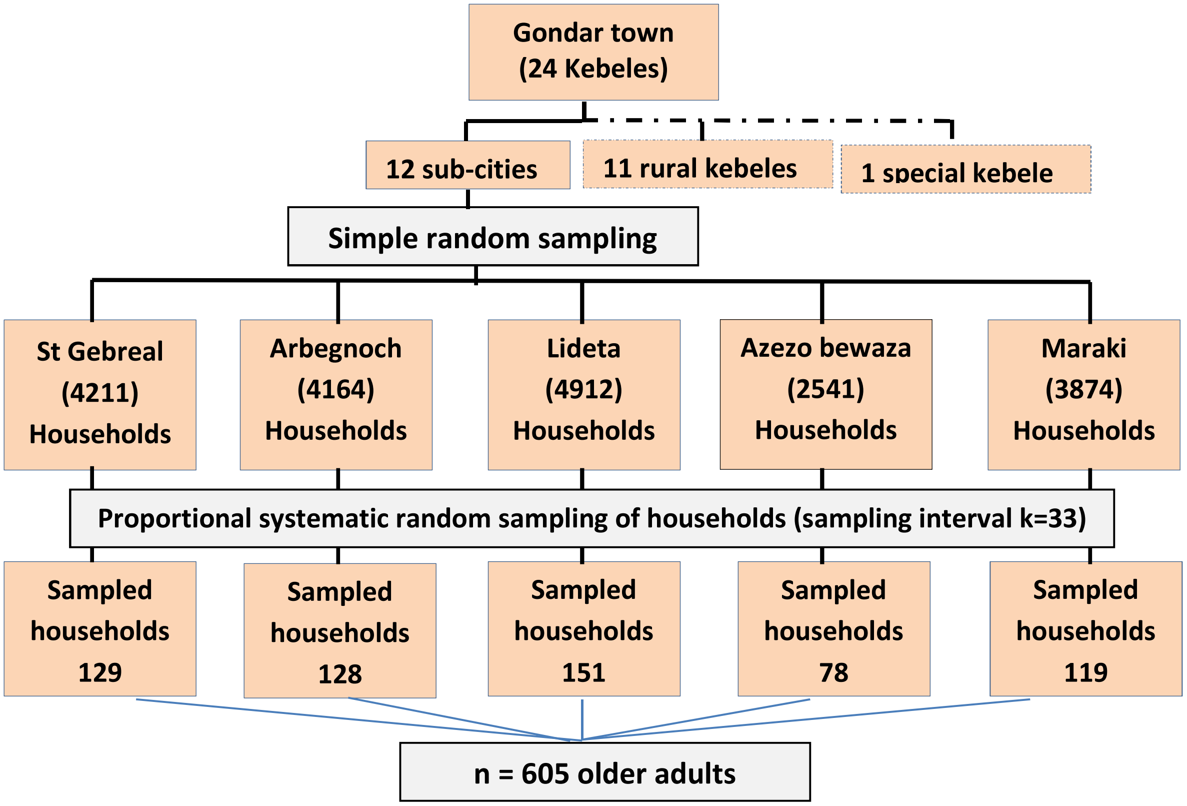

Supplement: S1 File — (PDF) [file pone.0221875.s001.pdf]
