# Supplementary material for: Falls among community-dwelling older adults in Ethiopia; A preliminary cross-sectional study
Source: PLoS One. 2019 Sep 10;14(9):e0221875. doi: 10.1371/journal.pone.0221875 (PMC6736232; doi:10.1371/journal.pone.0221875)
Supplement: S2 File — (DOCX) [file pone.0221875.s002.docx]

# Title: Falls among community-dwelling older adults in Ethiopia; A preliminary cross-sectional study

**ID: PONE-D-19-06676**

**STROBE** Statement—checklist of items that should be included in reports of ***cross-sectional studies***

|  | Item No | Recommendation |
| --- | --- | --- |
| **Title and abstract** | 1 | 1. Study design is indicated in the abstract, methods section as a community based cross-sectional study design. |
|  |  | (b) A balanced summary of what was done and what was found is provided in the methods and result section of the abstract |
| Introduction | | |
| Background/rationale | 2 | The scientific background and rationale for the investigation is reported. |
| Objectives | 3 | Specific objectives are stated as the final sentence of last paragraph under background session |
| Methods | | |
| Study design | 4 | It is presented in the first sentence of first para of method section |
| Setting | 5 | A detailed description of the study area, location, and population, estimate of households, terrain, and altitude are described in the first para of method section. |
| Participants | 6 | Eligibility criteria, the sources and methods of selection of participants are clearly stated in the last paragraph of methods session. |
| Variables | 7 | Both outcome and predictor variables are defined under Study variable sub-section in the methods in 1^st^ and 2^nd^ para. |
| Data sources/ measurement | 8 | Source of data and data analysis methods are discussed in the 5^th^ and 6^th^ paragraphs of methods session |
| Bias | 9 | Efforts to address potential sources of bias were described in several part of method session. |
| Study size | 10 | Study size determination and the flow of sampling was mentioned under sample size sub-session in method section and in addition a support file is also incorporated. |
| Quantitative variables | 11 | All quantitative variables treated as qualitative after categorizing them in one of most commonly used categories. |
| Statistical methods | 12 | (*a*) Statistical methods used in this study are described under data analysis sub-section in the last para of method session. |
|  |  | (*b*) Both sub group analysis and interaction terms were used. |
|  |  | (*c*) There were no missing data in this study |
|  |  | *(d)* Not applicable |
|  |  | (*e*) Not applicable |
| Results | | |
| Participants | 13 | (a) Number of participants/households, response rate is presented in the first paragraph of results session and detail socio-demographic characteristics and fall distribution in the table 1. |
|  |  | (b) About 99% participant responded and the most common reason for non-response is mentioned in the first para of result section. |
|  |  | (c) This was cross-sectional study so; there is no flow as that of longitudinal study. |
| Descriptive data | 14 | (a) Characteristics of study participants (eg demographic, clinical, social) and information on exposures and potential confounders is presented in tables 1 & 2 |
|  |  | (b) There were no missing data in this study |
| Outcome data | 15 | Outcome variable (fall in the past 12 months) described and summarized in table 2 and 3 |
| Main results | 16 | (*a*) Unadjusted estimates and confounder-adjusted estimates and their precision (eg, 95% confidence interval) are presented in table 4. Discussed under regression analysis 1^st^ para in result section. |
|  |  | 1. Category boundaries of continuous variables were categorized and reported in all tables. |
|  |  | (*c*) Regression model was used and expressed in odds ratio. |
| Other analyses | 17 | No clear or significant sub group difference noted and interaction terms were used but non-significant. |
| Discussion | | |
| Key results | 18 | Key results to study objectives are discussed under discussion session with references. |
| Limitations | 19 | Limitations and possible strengths related to the current study are discussed in the final paragraph of discussion session on the way of viewing direction for researchers. |
| Interpretation | 20 | A cautious overall interpretation of results considering objectives, results from similar studies, and other relevant evidence is discussed under limitation para of discussion session. |
| Generalisability | 21 | Generalisability (external validity) of the study results are mentioned under conclusion section |
| Other information | | |
| Funding | 22 | Information regarding the source of funding (University of Gondar) and the role of the funders for the present study is presented under acknowledgment section. |
